# Centering Ability of ProTaper Next and WaveOne Classic in J-Shape Simulated Root Canals

**DOI:** 10.1155/2016/1606013

**Published:** 2016-12-08

**Authors:** Giuseppe Troiano, Mario Dioguardi, Armando Cocco, Michele Giuliani, Cristiano Fabiani, Alfonso D'Alessandro, Domenico Ciavarella, Lorenzo Lo Muzio

**Affiliations:** Department of Clinical and Experimental Medicine, Foggia University, Foggia, Italy

## Abstract

*Introduction*. The aim of this study was to evaluate and compare the shaping and centering ability of ProTaper Next (PTN; Dentsply Maillefer, Ballaigues, Switzerland) and WaveOne Classic systems (Dentsply Maillefer) in simulated root canals.* Methods*. Forty J-shaped canals in resin blocks were assigned to two groups (*n* = 20 for each group). Photographic method was used to record pre- and postinstrumentation images. After superimposition, centering and shaping ability were recorded at 9 different levels from the apex using the software Autocad 2013 (Autodesk Inc., San Rafael, USA).* Results*. Shaping procedures with ProTaper Next resulted in a lower amount of resin removed at each reference point level. In addition, the pattern of centering ability improved after the use of ProTaper Next in 8 of 9 measurement points.* Conclusions*. Within the limitations of this study, shaping procedures with ProTaper Next instruments demonstrated a lower amount of resin removed and a better centering ability than WaveOne Classic system.

## 1. Introduction

A correct shaping is one of the most important goals to achieve success in endodontic treatment [1]. Ni-Ti instruments allow an easier way to prepare root canals and thanks to their elastic properties enable a reduction of the mean time of shaping [2]. Ni-Ti systems differ for taper, tip size, blade pitch, type of rotary motion, and instruments number. The widespread use of Ni-Ti rotary systems is based on Crown-Down concepts that allow a reduction of intracanal friction and should decrease the risk of intracanal fracture [3]. The fracture of the instrument may be caused by an incorrect use of the handpiece with an exaggerated pressure; it may occur when the instruments flex into the canal, alternating tensive and compressive cycles, increasing the cyclic fatigue; it may also be due to a taper lock because the noncutting tip size is larger than the cross section of the canal [4]. The possibility of taper lock may be decreased performing a glide path which allows an easier tool passage in the canals. Moreover, it is important to focus on the centering ability of the instruments, allowing a homogeneous shaping of the canal walls and decreasing the untouched areas at the end of the shaping procedures [5].

In the last years many new instruments were placed on the market and, among them, WaveOne (Dentsply Maillefer, Ballaigues, Switzerland) was introduced as a reciprocating system in 2011. It is a single-file system with a modified convex triangular cross section at the tip end and a convex triangular cross section, similar to ProTaper Universal, at the coronal end [6–8]. According to the manufacturer, the instrument should rotate at approximately 350 rpm with 30° of clockwise (CW) and 150° of counterclockwise (CCW) rotation angles [9, 10]. It has been demonstrated that single-file reciprocating systems result in a decreased time of shaping and in a similar maintenance of the original curvature of the canal when compared with conventional rotary systems [11]. More recently, ProTaper Next (PTN) (Dentsply Maillefer, Ballaigues, Switzerland), a novel set of Ni-Ti rotary instruments, has been introduced on the market. This set is composed of 5 instruments with different tip size and taper (at the tip of X1 #17/.04, #X2 25/.06, #X3 30/0.75, #X4 40/.06, and #X5 50/.06). PTN has an off-centered cross-sectional design and variable percentage of files tapers [12, 13]. Both these systems are manufactured with M-Wire alloy which allows a better flexibility and improves the resistance to cyclic fatigue [14]. The purpose of this study was to assess the possible differences in centering ability and overall postoperative shape of the canal with the use of ProTaper Next (asymmetric rotary motion) and WaveOne (counterclockwise reciprocating motion) in J-shape simulated root canals.

## 2. Materials and Methods

### 2.1. Shaping Procedures

Twenty J-shaped ISO 15 0.02 taper endo training blocks (Dentsply Maillefer) were assigned to two groups for a total of 40. Endoblocks belonging to groups 1 and 2 were shaped by an expert operator, postgraduate in endodontics, with more than ten years of experience. In both groups, the working length (WL) was assessed by a K10 file and the glide path was achieved with path files 1, 2, and 3 (Dentsply Maillefer) at the WL. In a second phase, samples of group 1 were shaped with a single WaveOne Primary reciprocating file to get in each block a tip size of 0.25 mm [9], while samples in group 2 were shaped with ProTaper Next files (X1 and X2). The files were mounted on a dedicated handpiece at the recommended setting suggested by the manufacturer for the handpiece (X-Smart, Dentsply Maillefer). In addition, in group 1, the WL was checked again when the WaveOne Classic Primary had reached the space between the middle and the apical third of the root canal [9]. Before the use, each instrument was lubricated with Glyde (Dentsply Maillefer) as a lubricating agent, and a rinse with 2,5% NaOCl was made after the use of each instrument. It is to note that a new set of instruments was used for shaping procedure of each simulated root canal.

### 2.2. Data Recording

**Figure 1:**
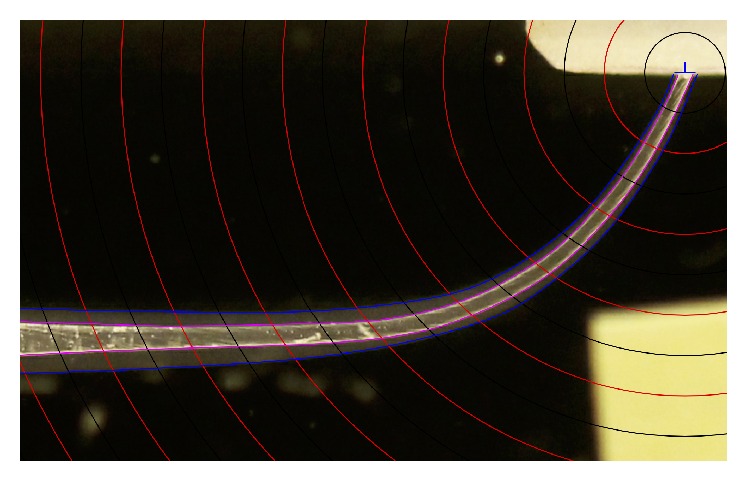
Reference points were built, constructing 9 concentric circles centering the apex at increasing diameters of 1 mm (red lines). To improve the accuracy of measurements, additional concentric circles were drawn starting at 0,5 mm from the apex at increasing diameters of 1 mm (black lines).

**Figure 2:**
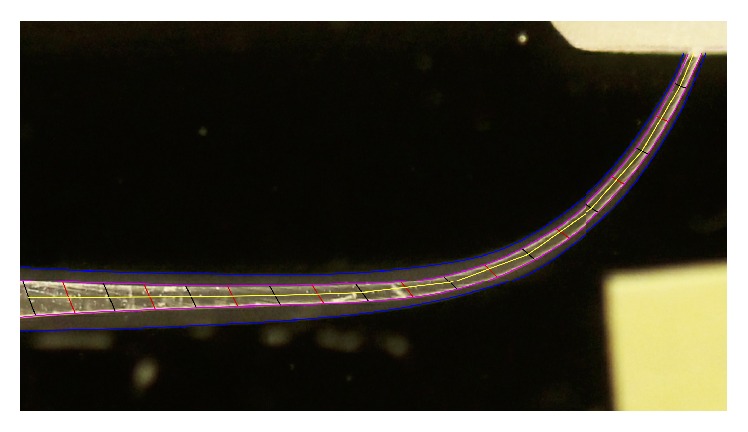
Arcs were obtained from the existing concentric circles (red lines) and from the additional ones (black lines); we also obtained the mean point of the chords subtended by each arch. A segmented straight line (yellow line), representing the mean preinstrumented axis, was therefore obtained joining together all the median points of the chords.

**Figure 3:**
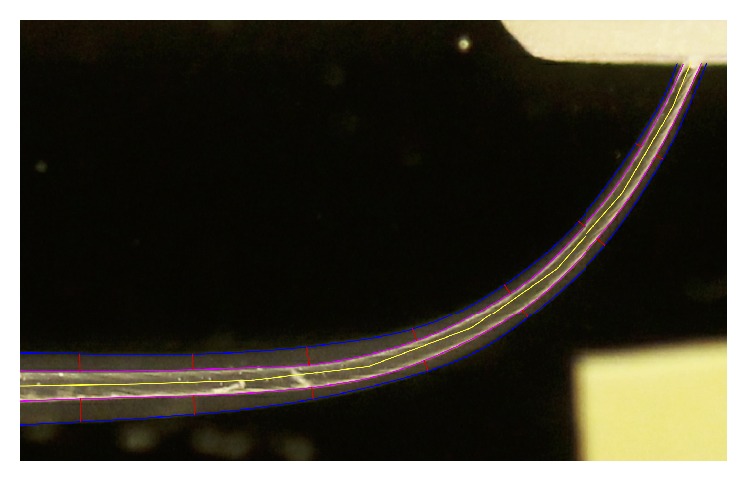
A perpendicular line to the segmented one was then drawn at each reference point. Afterwards, with the command “CUT,” two smaller fractions of the perpendicular lines comprised between the inner and outer limits of the canal, before and after shaping, were obtained.

Pre- and postinstrumentation images were recorded with a digital camera (Canon 1100D, Tokyo, Japan) at a fixed position and magnification using stable supports for digital camera and for specimens. Reference points were placed on the blocks to facilitate the subsequent superimposition, carried out with the use of dedicated imaging software (GIMP 2.8, Free Software Foundation, Boston, USA). Superimposed images were loaded on Autocad 2013 as raster image reference (Autodesk Inc., San Rafael, USA) to perform shaping analysis. The 9 level reference points were built after the construction of 9 concentric circles with center in the apex and increasing diameters of 1 mm. In our study, to improve the accuracy of measurements (mostly in the curvature region), additional concentric circles were drawn starting at 0,5 mm from the apex (Figure 1). Using the command “CUT” on Autocad, arcs were obtained from the existing concentric circles and from the new ones; with the command “MEAN SNAP” the midpoint of the chord subtended by each arch was obtained. A segmented straight line (mean preinstrumented axis) was therefore obtained joining together all the median points of the chords (Figure 2); using the command “UCS object,” a perpendicular line to the segmented one was then drawn at each reference point. Afterwards, with the command “CUT,” two smaller fractions of the perpendicular lines comprised between the inner and outer limits of the canal, before and after shaping, were obtained (Figure 3). This procedure allowed us to better evaluate the amount of resin removed accordingly to the curvature of the canal (18 measurements for each canal). Centering ability was then evaluated at each reference point subtracting the amount of resin removed from the inner part to that removed from the outer wall of the canal [15], while the overall postoperative shape was calculated adding these two measurements [16].

### 2.3. Statistical Analysis

Data have been analyzed using GraphPad Prism software 6.00 (GraphPad Prism Software, San Diego, CA, USA) by an expert in statistical analysis. Presence of normal distribution was assessed by Kolmogorov-Smirnoff test and probability plot graph. Statistical significance between different groups was determined with unpaired *t*-test; a level of *P* < 0,05 was considered to be statistically significant.

## 3. Results

### 3.1. Amount of Resin Removed

**Figure 4:**
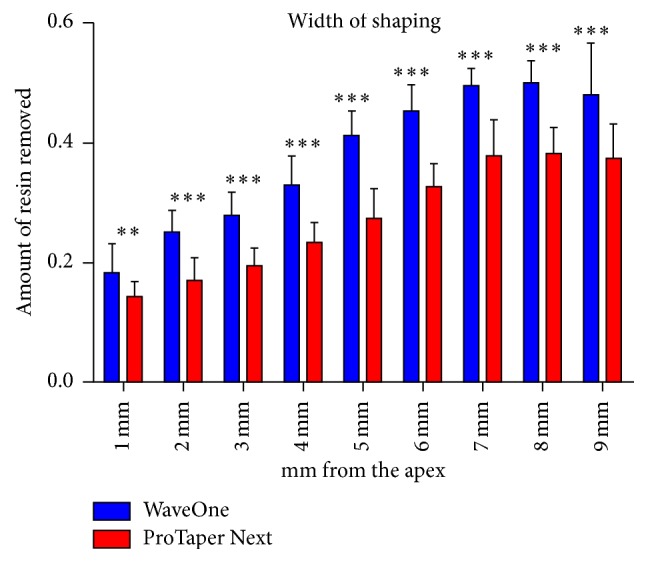
Amount of resin removed at 9-point level (^*∗∗*^ *P* < 0.01; ^*∗∗∗*^ *P* < 0.001).

No instrument fractured at the end of the shaping procedures. The amount of resin removed from the inner and outer wall of the canal was measured 1 to 9 millimeters from the apex and is summarized in Table 1. Adding the internal and external measurements for each millimeter, the total amount of resin removed was obtained for each measurement point (Figure 4). The difference between the two systems has been found significant in all the reference points.

### 3.2. Centering Ability

**Figure 5:**
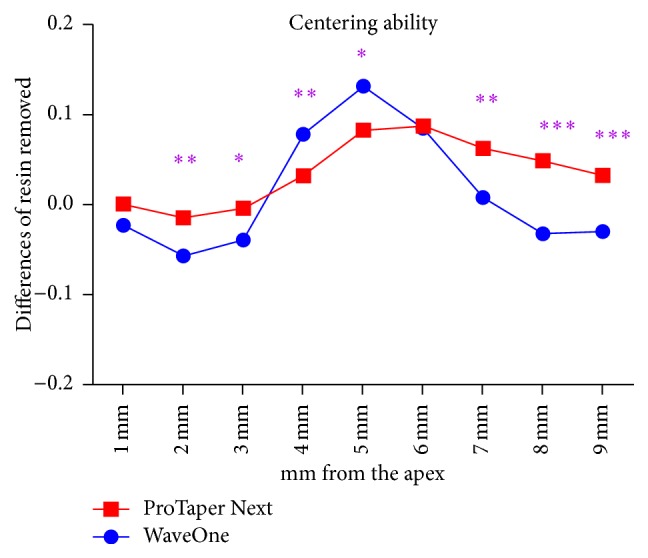
Centering ability in the four groups (^*∗*^ *P* < 0.05; ^*∗∗*^ *P* < 0.01; ^*∗∗∗*^ *P* < 0.001).

The centering ability was evaluated between groups and reported in Figure 5. The difference was statistically significant in 7 out of 9 levels. The pattern of centering ability seems to be more in favour of ProTaper Next than WaveOne Classic. Similarity has been found only at 1 and 6 mm from the apex (*P* > 0,05)

## 4. Discussion

The shaping of the root canal is one of the most important steps in endodontics, in order to obtain a pathway suitable for the subsequent action of irrigants and the subsequent root filling [3]. Thanks to the elastic properties of the alloy, the Ni-Ti instruments allow a simpler and easier use during the procedures of shaping. [17]. A wide range of instruments have been developed providing clinicians various options for a correct root canal shaping. These systems differ among themselves for cutting section, instrumentation sequence, subtype of Ni-Ti alloy and dynamics of motion. Until now, few studies directly compared shaping ability and centering ratio between a single-file reciprocating system (WaveOne) and a multifile asymmetrical rotational system (ProTaper Next). Both instruments are made with the new M-wire technology which, thanks to its nanocrystalline microstructure, determines a higher strength and wear resistance of these instruments [18, 19]. The study has been performed on simulated root canals that allow a direct visualization and represent a valid tool for comparative analysis [20]. They simulate a reliable reproduction of curved canals, which represent a hard challenge for clinical instrumentation [21]. The performance of these different instruments has been correlated with the capability to maintain the original canal anatomy [22]. Analysis on centering ability and width of shaping was performed after photographic superimposition, at 9 reference points from the apex [23]. As reported in previous studies, the glide-path procedures using path files could improve the shaping ability for both systems. In particular, performing the glide path has been demonstrated to reduce the number of pecking motions required to reach full WL when shaping with WaveOne Classic was performed [24]. Differences in width of shaping have been found at all nine levels, probably due to the different taper of the instruments (WaveOne 0.08, ProTaper Next 0.06). In a recent review, it has been supposed that “Instruments with an offset rotational mass may describe a larger envelope of motion than similarly sized files with symmetrical mass and axis of rotation” [25]; however the results of this study showed a better centering ability with ProTaper Next and are, hence, in disagreement with such statement. Our results could be influenced by the major apical transportation determined by the use of WaveOne system demonstrated in a previous study [26], although the absence, in this study, of differences at 1 mm from WL appears to refute this possibility. Through the analysis of centering ability, it is possible to evaluate the symmetry of shaping. This is very important during clinical shaping procedures to avoid formation of iatrogenic lesions [27]. In fact, if the instrument works more against one of the canal walls, this could cause stripping or other canal aberrations [28]. Previous analysis on centering ability did not find differences between ProTaper Next and other types of files, including the WaveOne [29]. However, in this study the centering ability of ProTaper Next resulted to be better in 7 out of 9 level points (Figure 2). Attention should be paid to the score of centering ratio which never exceeded 0.1 mm. The possible differences among these results could be due to different methodologies and models. However, the results of this study indicate a clear improvement in centering ability of ProTaper Next when compared with WaveOne Classic.

## 5. Conclusions

Within the limitations of this study, it could be concluded that ProTaper Next (X1, X2) might determine a lower amount of resin removed and a better centering ability compared with WaveOne Classic Primary.

## Figures and Tables

**Table 1:** Analysis of the amount resin removed from the inner and outer aspect of the canal at nine-point level from the apex.

	Inner canal side (mm from the apex)									Outer canal side (mm from the apex)								
	1	2	3	4	5	6	7	8	9	1	2	3	4	5	6	7	8	9
Wave One																		
Mean	0.080	0.097	0.119	0.204	0.272	0.269	0.251	0.234	0.224	0.102	0153	0.158	0.125	0.139	0.183	0.242	0.265	0.253
SD	0.028	0.021	0.027	0.041	0.048	0.048	0.036	0.031	0.044	0.036	0.033	0.027	0.025	0.028	0.043	0.040	0.037	0.054
ProTaper Next																		
Mean	0.072	0.078	0.095	0.132	0.178	0.207	0.220	0.215	0.203	0.071	0.092	0.098	0.099	0.095	0.119	0.157	0.166	0.169
SD	0.023	0.022	0.025	0.034	0.056	0.038	0.037	0.035	0.042	0.017	0.033	0.030	0.025	0.020	0.030	0.046	0.030	0.034

*P* value	—	*∗∗*	*∗∗*	*∗∗∗*	*∗∗∗*	*∗∗∗*	*∗*	—	—	*∗∗*	*∗∗∗*	*∗∗∗*	*∗∗*	*∗∗∗*	*∗∗∗*	*∗∗∗*	*∗∗∗*	*∗∗∗*

^*∗*^ *P* < 0.05; ^*∗∗*^ *P* < 0.01; ^*∗∗∗*^ *P* < 0.001.
